# Supplementary material for: Development of medical informatics in China over the past 30 years from a conference perspective and a Sino-American comparison
Source: PeerJ. 2017 Nov 21;5:e4082. doi: 10.7717/peerj.4082 (PMC5701558; doi:10.7717/peerj.4082)
Supplement: Supplemental Information 1 [file peerj-05-4082-s001.doc]

**Appendix A**

**A-1 Evolution of Hospital Information Systems in China**

Hospital information systems in China emerged in the 1970s and developed at the end of the 1990s, but hospital information construction used to be regarded by many hospital managers as dispensable. The end of the 1990s witnessed the No.1 Military Medical Informatics Project in the army [1] and the China Hospital Information System (CHIS) by Beijing Zhongbang Huizhi Company in the public sphere [2], both of which were extensively applied in the medical industry, thus solving many problems that could not be handled by manual management and bringing about the preliminary popularization of hospital information construction. After 2010, an explosive development occurred due to the policy guidance specified by the new medical reforms.

The development of hospital information systems in China can be divided into three stages.

**Stage one: solitary application (1979-2002)**

This stage witnessed the appearance of some single-computer and single-task systems, including clinical management, drug management, cost management, equipment management, and storeroom management. This stage was characterized by the interdependence between systems and unshared information.

**Stage two: department-level application (2003-2009)**

This stage witnessed the charge systems, outpatient service systems, integrated drug management from storeroom to drug shops, patient registration centered on medical record first-pages and medical statistics management systems. These systems met the department-level demands as a whole. The 2010 CHIMA China hospital information construction statistics show that the rates of HIS are 30%-35% in most regions, ranging from 80% in East China to only 20% in Northwest China, and are up to 84% in provincial hospitals. At this stage, HIS was applied to different aspects of subsystems but was focused on charges. Moreover, information sharing was achieved within subsystems but not between subsystems.

**Stage three: hospital and region integrated information system construction (2010-now).**

At this stage, information integration at the hospital level has been generally achieved. The overall frame structure of different links around patients’ in-hospital activities has been established based on EMR. Information sharing between different systems has been established to form a closed-loop CIS. Additionally, the interconnection and interworking of hospital EMR and regional EHRs have been completed. However, the development of hospital information systems in China still lags behind the developed countries and is regionally unbalanced. In the flourishing coast areas of East China, First-class Hospitals at Grade 3 have completed EMR construction and preliminarily realized the interoperation of in-hospital EMR and regional EHRs in clinical diagnosis, diagnosis & treatment, medication, and examination & inspection. Nevertheless, the majority of hospitals in West China are still under HIS construction.

**A-2 Evolution of Chinese Mainstream Medical Informatics Conferences**

**CMIA AND CMIAAS**

The China Medical Informatics Association (CMIA) is an academic team of experts, technical staff and management staff applying information science and technology to the medicine and health care fields and was established in 1980 under the leadership of the Ministry of Industry & Information Technology, Ministry of Science & Technology, Ministry of Education, National Health & Family Planning Commission, Medical Department of the General Logistics Department, China Food & Drug Supervision Administration, and State Administration of Traditional Chinese Medicine [3]. CMIA is China’s only representative in the International Medical Informatics Association (IMIA) for international communication and cooperation, and, as the Chinese committee of IMIA, it successfully held MEDINFO’89 [4]. CMIA will hold the 16th IMIA World Medical Informatics Conference MEDINFO’2017 in Hangzhou.

Since its establishment in 1980, CMIA has held 13 China Medical Informatics Association Annual Symposiums (CMIAAS) (triennial) [5], carried out state-wide academic exchanges and published 15 collections. Since the first CMI at Changsha in 1981, the 13th CMI at Shanghai in 2015 was attended by academicians and famous scholars Prof. Longde Wang and Prof. Qimin Zhan, who gave reports about the frontiers of medicine informatics. Corresponding leaders from the Informatization Office of State Council, National Health & Family Planning Commission, and Institute of Social Development and Academy of Macroeconomic Research, both from the National Development & Reform Commission (NDRC), thoroughly introduced some policies about medical big data and telemedicine. More than 200 representatives from different provinces, autonomous regions, and municipalities under direct jurisdiction, the People's Liberation Army circle, and well-known Chinese companies attended the conference. There were five forums, including "Electronic Medical Records and Hospital Informatics," "Regional Health Information System," "Telemedicine Health Big Data," "Medical Information Education, Medical Signals and Image Processing," and "Research and Application of Nursing Informatics." A total of 71 papers were included.

**CHIMA AND CHINC**

The China Hospital Information Management Association (CHIMA) was established in 1996 and affiliated with the Chinese Hospital Association. The objectives of its conferences are to: promote academic exchange activities in hospital information management between China and foreign countries; formulate hospital information management standards and regulations; train hospital information management staff with improved quality; and promote the development of hospital information management works in China [6]. CHIMA has organized and published a medical informatics textbook *Information Management Manual for Hospital Management* and aftermarket investigations into hospital information systems and electronic medical record products in China, and has also released reports and white books *Case Studies on Chinese Hospital Information Systems*, *HIS Performance Investigation*, and *Investigation into Electronic Medical Record Market Mainstream Products*. Its official journal is *the China Digital Medicine.*

After its establishment, CHIMA has jointly held the China Hospital Information Network Conference (CHINC) every year with the National Institute of Institute of Administration [7]. Since the first conference at Beijing in 1997, there are now 20 conferences (annual). The "2015 China Hospital Information Network Conference and Hospital Information Forum across Taiwan Straits" was held in Xiamen. Prof. Zheng Fu (former undersecretary of the Medical Department of the General Logistics Department), Prof. John Frenzel (secretary of the Australian Medical Information Association) and Prof. Jiajie Zhang (head of the School of Biomedical Informatics, University of Texas Health Science Center at Houston) attended the opening ceremony. More than 3000 experts and representatives and over 150 medical informatics companies at home and abroad joined the conference. There were seven forums, including "Director Forum across Taiwan Strait," "China and U.S. Informatics Forum," "Nurse Informatics Forum," "Private Hospital Informatics Forum," "Internet + Medical Treatment," "Youth Forum," and "Clinical Data Center." The themes of HIT application were international/domestic hotspots of information development, electronic medical records, nurse information development, mobile medicine & optimized patient service, and integrated platform & data center construction. This conference collected 853 papers and selected 39 excellent papers through its screening process.

**CHIA AND CHITEC**

The Chinese Health Information Association (CHIA) is a national first-level association administered by the National Health & Family Planning Commission. Its predecessor was the China Health Statistics Association [8], which was formally established in September 1984 in Nanning, Guangxi Zhuang Autonomous Region, and renamed in June 2004. CHIA has 17 specialized committees that cover electronic medical records & hospital informatics, medical statistics education, public health informatics, telemedicine informatics, and Chinese herbal medicine informatics. Its official journal is the *Chinese Journal of Health Informatics and Management*.

Since its renaming in 2004, CHIA has held 12 annual conferences (Chinese Health Information Technology Exchange Conference, CHITEC). The "2015 CHITEC and Electronic Health Forum from Mainland China, Taiwan, Hong Kong, Macau" was held in Suzhou [9]. More than 3000 participants and over 100 international and domestic companies were present, including leaders and specialized technical staff from different medical and health institutions in China; experts and scholars from different colleges, universities and institutes across China; experts and scholars from Hong Kong, Macau and Taiwan; health information management and technical staff from WHO West Pacific members; and CIOs and technical staff from well-known foreign and domestic IT companies. Dr. Xiaohong Chen (deputy director of the National Health and Family Planning Commission), Dr. Hede Shi (representative of WHO in China) and Dr. Jiehong Hua (first counsellor for health and consumer affairs from the European Union delegation in China) attended the conference. The opening ceremony was hosted by Prof. Qun Meng, director at the Statistics Information Center of MOH. There were ten themed forums, including "electronic medical records & clinical data center," "regional health information development and business collaboration," "health statistics and big data," "information beneficial project & health card application," "public health and intelligent health management," "E-government and information security," and "telemedicine and digital medical equipment." This conference collected 667 papers and selected 37 excellent ones by screening. The themes covered seven HIT application fields, including hospital informatics, health statistics, health informatics, one-card solution, mobile medicine, and telemedicine.

**SCMI AND CPMI**

The Society of Chinese Medical Information (SCMI) is a branch of the Chinese Medical Assistance CPMI and was established in Nanjing, Jiangsu Province, in 1993 [10]. It initially aimed to promote the development of medicine books and information affairs in China and cultivate novel medical book and informatics staff; later, it gradually began to pay attention to academic research about medical health informatics and medical information. Its official journal is the *Chinese Journal of Medical Library and Information Science*.

The 21st China Proceedings of Medical Informatics (CPMIs) have been held annually since the first one was held in Nanjing in June 1994[11]. The 21st CPMI was held in 2015 in Zhengzhou, Henan province. Nearly 300 participants from medical information institutions, medical specialty libraries, and hospital information departments attended. The opening ceremony was hosted by Prof. Rui Chen, deputy director and secretary of SCMI and director of the Medical Library of Chinese PLA. It collected 248 papers, including 13 conference exchange papers and 48 sub-conference exchange papers. The themes covered "medical information and information retrieval," "hospital information development," "medical information resource construction and services," and "medical information education and personnel training." There were four sub-forums, including "Medical Informatics Theory & Practice and Medical & Health System Reform," "Health Information and Medical Informatics Personnel Training," "Library Resource Construction and Information Service," and "Youth Academic Salon."

**A-3 Unique Characteristics of the Medical Informatics Discipline in China**

**Weak foundation**

The early phases of MI development in China were dominated by library science and information science. At present, SCMI is still an academic institution focused on medical libraries and medical information searching, but the discipline of MI has lagged behind, owing to the lack of support from computer science and information technology. At the early stages (2003-2009), the major service entities of MI in China were medical institutions, and over 80% of medical resources were controlled by public hospitals [12]. The functional departments of public hospitals used MI to support the execution of governmental function policies, management in medical institutions, and provision of research data. As a typical application in clinical medicine, Chinese HIS was very cost-centered and acted for medical management. At this stage, there was a trend to integrate MI with management, information intelligence and medical statistics and to facilitate hospital management, health reform policy implementation, public health management, and statistical analysis of scientific data. This trend guided the MI research and HIT application in China, which were epitomized in the conference collections. Specifically, the absolutely dominant themes were "hospital information development" and "regional, public and grass-root health informatics and telemedicine." Compared with the global trend to integrate MI and bioinformatics (BI) into Biomedical Informatics (BMI) [13-16], BI in China tended to combine with biomedical engineering, while public hospitals set up medical equipment maintenance departments exclusively for biomedical engineering. Moreover, the IT departments in Chinese hospitals considered MI as a secondary discipline of biotechnique, rather than an independent discipline. Some IT departments were even affiliated with medical equipment maintenance departments. This phenomenon of confusing mainstream MI with biomedical engineering or library science or information retrieval is very common, especially in CPMI.

**Lack of discipline construction**

Compared with the global definition of MI subfield classifications, the MI discipline in China is limited by incomplete construction and incomplete specialty setting. A few of the 5086 included papers were focused on "medical information theory," "biomedical cognition science," or "standards, safety and laws." There were no papers about "consumer health information," "bioinformatics," "robots, virtual medical treatment," or "individual health records and self-health care system."

**Theoretical research fails to support practical application**

Due to the weak theoretical basis, there is little theoretical or technical research on MI. Only a few teaching institutions have set up MI institutes or graduate courses (27 master's degree and 5 doctoral degree conference places), but most students are undergraduates [17]. The academic circle cannot provide enough theoretical support for the industry and pays little attention to research & development of basic support facilities or specialized tools. There were 214 (4.2%) conference papers focused on "medical information theories and education" compared with the dominant themes about health care and population health informatics application. The industrial field prefers to develop tools on and to apply the medical management in a specific medical business scene using conventional software. This phenomenon of focusing "more on application in HIT and less on research on MI" has led to severe repeated HIT construction and huge resource waste. Moreover, very little investment from the Chinese government has been devoted to basic MI research compared with the huge investments into HIT application, especially medical informatics. We searched and compared the medical information projects funded by the Natural Science Foundation of China (NSFC) [18]. As the main channel from the Chinese government to support basic and applied basic research, NSFC is targeted at the whole country as well as researchers from universities, colleges, research institutions, and enterprises. The project application codes in the field of NSFC were very concentrated. One application code related to independent medical information has been established since 2005 (code: H1814, medical information system and telemedicine). So far, it has funded 19 projects, approximately 1.7 projects annually, and the average fund sum was 47,900 US dollars /project. This number was only 52.8% that of "nuclear medicine" (code: H1806) under the same code, H1806 (which funded 36 projects in 2015), in January 2015; moreover, the average sum per project was 61% that of the latter 78,600 US dollars)/project.

**The academic circle is unable to provide qualified applied personnel for the industrial field**

So far, the industry does not highly approve of the personnel produced by academic circles. As one major representative in the industry, hospital IT centers lack comprehensive talents majoring in medicine and informatics [19]. Few workers, even those in hospital IT departments, have received formal high-quality MI academic training. The CHIMA 2010 *China Hospital Informatics Development Report* (white book) shows [20] that the IT professional staff in superior third-class hospitals in China ranges from 3-30, and IT professionals from only a very few hospitals are postgraduates or above; most of them have only a master's degree or below. These workers mostly major in computer-related specialties, but very few have graduated from medicine-related majors. Analysis of the 2005 American Medical Informatics Association (AMIA) Annual Symposium shows that authors with titles in medical majors contributed 80% of the 158 papers [20]. Moreover, the hospital IT human resources are significantly different between China and the U.S. Analysis of full-time IT workers regarding hospital informatics, conducted by U.S. HIMSS in 2006, shows that 80% of hospitals maintain at least 10 IT workers, and 31% of hospitals have more than 50 IT workers, while the data were 77% and 1%, respectively, in China [20]. This situation of staff composition has led hospital IT departments to prefer technical selection and the strategies supported by “hospital informatics,” namely, "mainly outsourcing." The hospital IT workers are only responsible for systematic-type selection and later maintenance. This situation aggravates the continuous contradiction between the demand for applied personnel and the supply of traditional medical information graduates. Furthermore, the hospitals have continued to ignore MI and view MI as replaceable and irrelevant, which hinders the disciplinary construction and development.

**A-4 Several Suggestions for the Development of the Medical Informatics Discipline with Chinese Characteristics**

**To set up state-level medical information disciplinary development targets and strategies.**

In the current medical field, medical informatics is regarded as a "better-to-have" rather than a "compulsory" discipline. It is, at most, considered as an assistant tool of administrative management but without clear development targets. Together with the population health informatics [21] and national development strategy on health care big data applications [22] strongly supported by Premier Keqiang Li, and under the overall plan for national medical information development, we should formulate an overall target of reasonable and definite discipline development and set up a complete discipline construction and basic setting support system.

**Based on the latest version of the IMIA education international manual [23, 24], we should put forward medical informatics personnel quality demands and profession frameworks that apply to China's actual conditions.**

The higher education on medical informatics in China has largely inherited the disciplinary characteristics of library science and philology. However, the personnel trained so far do not cater to the HIT field and are far less qualified regarding real knowledge and technology in the industrial field. Taking hospital IT centers as an example, the staff mainly majors in computer science. Thus, medical and health informatics personnel development strategy, scientific planning medical & health system informatics construction personnel demands, specialty divisions, knowledge & skill demands, training plans, and examination systems jointly formulated by the National Health & Family Planning Commission and the Ministry of Education are needed.

**CMIA, CHIMA, CHIA and SCMI should proactively undertake international collaboration and research, according to the demands of MI disciplinary development in China and following international research fields.**

Through "inviting inside, sending out, teacher exchange and student exchange," these organizations should improve teaching and academic abilities and eliminate the differences between the international overall developing levels and those in China. As state-level academic institutions, they should play a leading role in the discipline. They should actively organize international collaborations and exchanges, send professionals outside for advanced studies and inspection, create international cooperation projects and introduce advanced philosophy and techniques.

**REFERENCE:**

[1] Hua-cai LI, Heng-nian CHEN, WANG Jing-ming, Xiao-gan SU. To Set up Information oriented Hospitals through“No 1 Military” Project. Hosp Admin J Chin PLA. 2000;7(6):409-10. (in Chinese)

[2] huai-cheng LI. Some experience of hospital information and network construction. Chin Hosp Mang. 1999;19(1):40-1. (in Chinese)

[3] China Medical Informatics Association. [cited accessed on 6.30.2016 (in Chinese); Available from: http://www.cmia.info/cn/details.asp?id=1

[4] MEDINFO 89. Beijing: China and Singapore: Republic of Singapore: IOS Publisher; 1989.

[5] China Medical Informatics Conference. [cited accessed on 6.30.2016 (in Chinese); Available from: http://www.cmia.info/cn/news_detail.asp?id=11902

[6] China Hospital Information Management Association. [cited accessed on 6.30.2016 (in Chinese); Available from:
http://www.chima.org.cn/index.php?m=content&c=index&a=lists&catid=6

[7] Chinese Hospital Informatics Network Conference [cited accessed on 6.30.2016 (in Chinese); Available from: http://www.chinc.org.cn/

[8] Chinese Health Information Association. [cited accessed on 6.30.2016 (in Chinese); Available from:
http://www.chia-moh.org.cn/zgwsxxxh/xhjj/index_lmtt.shtml

[9] Chinese Health Information Technology Exchange Conference. [cited accessed on 6.30.2016 (in Chinese); Available from: http://www.chia-moh.org.cn/zgwsxxxh/zxdhxxn/201507/c2dfca8fc12c495696761895d721d8ae.shtml

[10] Chinese Society of Medical Information. [cited accessed on 6.30.2016 (in Chinese); Available from: http://csmi.cma.org.cn/xxxfh_about/index.html

[11] China Proceedings of Medical Informatics [cited accessed on 6.30.2016 (in Chinese); Available from: http://csmi2016.medmeeting.org/cn

[12] Jiang-Nan Cai. Socialization of medical resources is the only way of Chinses medical reform. Chin J hosp lead deci. 2013;13(1):1-3. (in Chinese)

[13] Martin-Sanchez F., Maojo V., Lopez-Campos G. Integrating genomics into health information systems. Methods Inf Med. 2002;41(1):25-30.

[14] Maojo V., Kulikowski C. A. Bioinformatics and medical informatics: collaborations on the road to genomic medicine? J Am Med Inform Assoc. 2003 Nov-Dec;10(6):515-22.

[15] Maojo V., Kulikowski C. Medical informatics and bioinformatics: integration or evolution through scientific crises? Methods Inf Med. 2006;45(5):474-82.

[16] Haux R. Medical informatics: past, present, future. Int J Med Inform. 2010 Sep;79(9):599-610.

[17] Li Xiaotao, Liu Yan, Liu Yamin, eee. Research heat-spots published indifferent periods of time of the two major medical library and informationscience journals of China: a comparative analysis. Chin J Med Lib Info. 2011;20(1):10-4. (in Chinese)

[18] The Natural Science Foundation of China. [cited accessed on 6.30.2016 Available from: http://www.nsfc.gov.cn/publish/portal1/

[19] Kannry J., Sengstack P., Thyvalikakath T. P., et al. The Chief Clinical Informatics Officer (CCIO): AMIA Task Force Report on CCIO Knowledge, Education, and Skillset Requirements. Applied clinical informatics. 2016;7(1):143-76.

[20] Li. BaoLuo. China Health White Paper. In: Li B, editor. China Health Information Technology Conference & International Forum. HangZhou: CHIMA; 2010.

[21] National Health and Family Planning Commission of China on Accelerating the Construction of Population Health Information Guidance. 2013 [cited accessed on 6.30.2016 (in Chinese)]; Available from: http://www.nhfpc.gov.cn/guihuaxxs/s10741/201312/09bce5f480e84747aa130428ca7fc8ad.shtml

[22] Chinese Promote and Standardize Health Care Data Application Development Guidance . 2016 [cited accessed on 6.30.2016 (in Chinese)]; Available from: http://www.gov.cn/xinwen/2016-06/25/content_5085401.htm

[23] Mantas J., Ammenwerth E., Demiris G., et al. Recommendations of the International Medical Informatics Association (IMIA) on Education in Biomedical and Health Informatics. First Revision. Methods Inf Med. 2010 Jan 7;49(2):105-20.

[24] Haux R., Murray P. J. On IMIA's International Activities in Health and Biomedical Informatics Education. Methods Inf Med. 2010;49(3):305-9.
